# Radiation dose has no significant impact on CT-based bone mineral density measurements in a large-animal model

**DOI:** 10.1038/s41598-026-55169-6

**Published:** 2026-05-28

**Authors:** Johannes Christian Harmes, Mathias Holtkamp, Jannis Straus, Gregor Jost, Michael Forsting, René Hosch, Felix Nensa, Hubertus Pietsch, Luca Salhöfer, Johannes Haubold

**Affiliations:** 1 Institute of Diagnostic and Interventional Radiology and Neuroradiology, University Hospital Essen, Essen, Germany; 2 Institute for Artificial Intelligence in Medicine, University Hospital Essen, Essen, Germany; 3 Bayer AG, Berlin, Germany

**Keywords:** Bone mineral density, Computed tomography, Radiation dose, Osteoporosis, Opportunistic screening, Machine learning, Biomarkers, Diseases, Medical research

## Abstract

Bone mineral density (BMD) is a biomarker for frailty, and CT-derived radiodensity can be extracted fully automatically as a surrogate. Because these measurements might be affected by image noise, which varies substantially in the clinical routine, this systematic large-animal study investigates the consistency of CT-based BMD measurements under different radiation dose settings. Twenty Göttingen minipigs underwent six non-contrast CT examinations with five dose levels (CTDIvol: 0.53–10.01 mGy; 5%, 10%, 20%, 40%, 100%; 600 scans). BMD was assessed using CT-derived radiodensity (Hounsfield units, HU) by segmenting the complete ninth thoracic vertebra, and by placing a region of interest (ROI) in the trabecular bone. RM-ANOVA was used to assess statistical significance. Data are presented as mean with standard deviation. The BMD measurement remained consistent between the control and the different dose settings. Even the lowest dose setting (5%: complete = 761 [± 56] HU, ROI = 749 [± 72] HU) showed no significant differences compared to the control (complete = 756 [± 55] HU, ROI = 738 [± 68] HU). Finally, CT-based BMD measurements remained consistent and are therefore robust to substantial dose reduction, indicating the technical feasibility of comparing CT examinations with different dose protocols, relevant for opportunistic screening.

## Introduction

Computed tomography (CT) is an integral part of modern medical diagnostics. Advancements in machine learning (ML) technologies have further enhanced the capabilities of CT, especially concerning volumetry of body tissues and organs^1^. Thereby, body composition analysis (BCA) has evolved from manual and semi-automatic segmentation to a fully automated 3D analysis^2^. These improvements lead to a great interest in the relationship between clinical outcomes and certain body composition parameters, which have been investigated in several disease conditions^3–8^.

Bone mineral density (BMD) is a well-established biomarker for frailty, survival, progression-free survival, and disease severity^9–14^. Osteoporosis leads to an increased risk of low-impact fractures which are associated with increased mortality and a substantial economic burden^15–17^.

Dual-energy X-ray absorptiometry (DXA) is the reference standard for BMD assessment^18^. It is a non-invasive, fast and low-dose method^19^. However, the two-dimensional data limit volumetric bone assessment^15,19,20^. Furthermore, DXA examinations require additional, albeit low, radiation exposure and lead to additional costs for limited information gain^19^. Therefore, after more than three decades of use, DXA continues to fall short of clinical demands^15^. Quantitative computed tomography (qCT) is an alternative to DXA scans because it provides a three-dimensional, more accurate BMD measurement but usually requires additional equipment in form of a phantom^15,18–22^.

Precise segmentation of the entire spine employing machine learning algorithms is the backbone for fully automated CT based BMD measurement via analysis of the radiodensity without the necessity of a phantom^21,23–25^. This is particularly interesting due to the general increase in the number of CT examinations (in the United States: 84.5 million in 2021 vs. 34.9 million in 2000) with an associated potential for detecting osteoporosis in routine imaging in form of opportunistic screening^15^.

However, before large-scale implementation, it is important to consider that different CT scanners and different dose protocols are used in everyday clinical practice^26–29^. Previous studies investigated the influence of radiation dose on CT-based BMD measurement in phantom-based qCTs and others in phantomless approaches and have concluded that ultra-low-dose (ULD) CTs achieve substantial dose reduction (85-92.5%) while preserving the diagnostic accuracy for osteoporosis classification^22,30^.

This systematic large-animal study investigates the robustness of CT-based BMD measurement under different radiation dose settings intra-individually in a controlled animal model.

## Materials and methods

### Ethics declaration

The study was approved by the State Animal Welfare Committee (Landesamt für Gesundheit und Soziales, Berlin, Germany), carried out in compliance with the German Animal Welfare Act and under consideration of the ARRIVE guidelines.

### Animals

Twenty Göttingen minipigs (Ellegaard Dalmose, Denmark) served as subjects in the study. After intramuscular injection of ketamine (15 ml/kg [Pharmacia, Karlsruhe, Germany]) and azaperone (2 mg/kg [Stresnil; Elanco GmbH, Bad Homburg, Germany]) followed by the intravenous administration of 7 mg/kg propofol (Propofol-Lipuro; B. Braun, Melsungen, Germany), the animals were orally intubated and mechanically ventilated with an air-oxygen mixture. Anesthesia was maintained with an intravenous propofol infusion of 12 mg/kg/h. The vital parameters of the animals were continuously monitored. This study is an additional investigation of parts of a previously conducted study^31,32^.

### Computed tomography imaging

The CT examinations were performed in the prone position and with end-expiratory ventilation stopped during the image acquisition on a 192-slice dual-source CT scanner (Somatom Force, Siemens Healthineers, Erlangen, Germany). The following uniform scan parameters were applied: 0.5 s rotation time, 0.6 pitch, 150 mm scan length, 90 kV tube voltage. The tube current was modified among the groups resulting in different tube current-time products: 20 mAs, 35 mAs, 70 mAs, 140 mAs, and 350 mAs (Control). Image reconstruction was performed with a 1-mm slice thickness, a 300 × 300 mm field of view, a Br40 kernel, and SAFIRE 3 iterative reconstruction (Table 1).

**Table 1:** Scan parameters.

Scan parameter	5%	10%	20%	40%	Control
Rotation time (s)	0.5	0.5	0.5	0.5	0.5
Pitch factor	0.6	0.6	0.6	0.6	0.6
Scan length (mm)	150	150	150	150	150
Tube potential (kV)	90	90	90	90	90
Tube current-time product (mAs)	20	35	70	140	350
CTDIvol (mGy)	0.53	0.96	1.98	4.00	10.01

mAs = milliampere second; s = second; mm= millimeter; kV = kilovolt; CTDIvol = volumetric CT dose index; mGy = milligray.

### Study design

Twenty Göttingen minipigs were subjected to six CT examinations of the epigastrium (repetitions) for a total of 120 examinations. Five non-contrast scans (5%, 10%, 20%, 40%, Control) were performed in each examination, for a total of 600 scans. Due to incomplete scans, three exams were removed from the study before statistical analysis, resulting in a total number of 117 examinations consisting of 585 scans.

### Fully automated BMD measurement

**Fig. 1:**
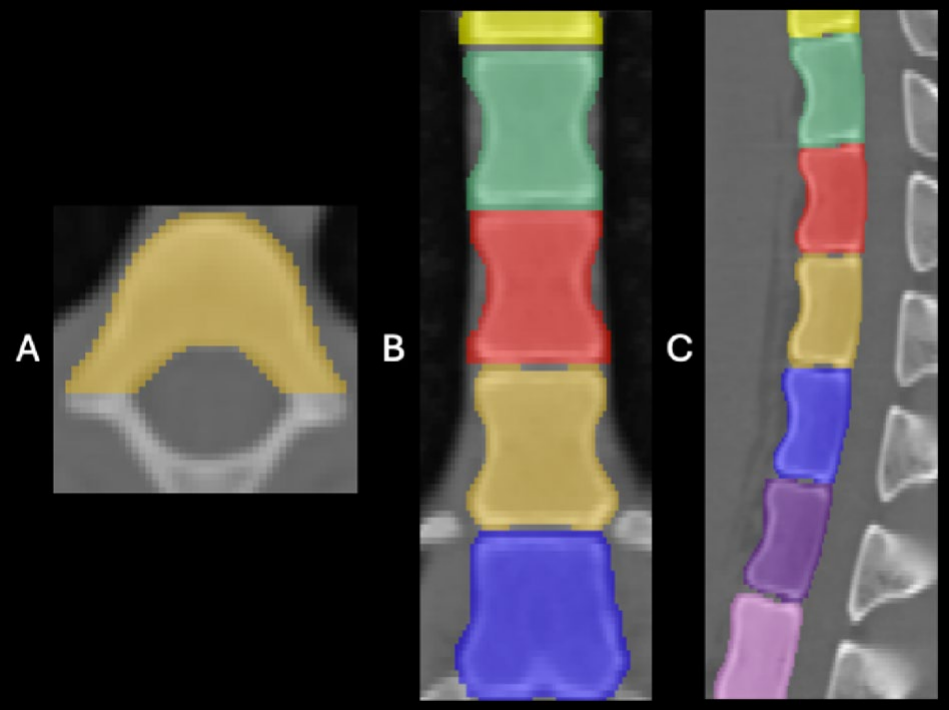
Segmentation of the vertebral bodies of a Göttingen minipig in a non-contrast CT examination with a radiation dose of 10.01 mGy in **A** axial, **B** coronal and **C** sagittal view. Th9 is annotated in brown. mGy = milligray.

The BMD measurement was performed by a fully automated, phantomless, volumetric, three-dimensional assessment of the mean radiodensity (Hounsfield units (HU)) of the ninth thoracic vertebral body (Th9) as a surrogate marker rather than absolute volumetric BMD values. Accordingly, all reported BMD values represent CT attenuation-derived surrogate measurements. No calibration phantom was used. The reason for the shape of the segmentation was the DXA scan as the reference standard for BMD assessment which measures the density of the entire vertebral body. A previously published network “Total Segmentator” within the framework of the “Body and Organ Analysis” (BOA) employing a multi-resolution variant of the nnU-Net architecture was utilized as a basis to extract the BMD^1,2,33^. This network facilitates automated BMD measurement in CT scans, already exhibiting high segmentation accuracy for the vertebral bodies^2^. To further enhance the 3D model to the anatomy of the Göttingen minipigs, each individual image slice of 13 randomly selected Göttinger minipigs (1950 slices) was manually segmented under the supervision of a board-certified radiologist (ten years of experience) and an extended nnU-Net 3D model was trained with three segmentation masks per scan by an experienced segmentation specialist (Fig. 1).

**Fig. 2:**
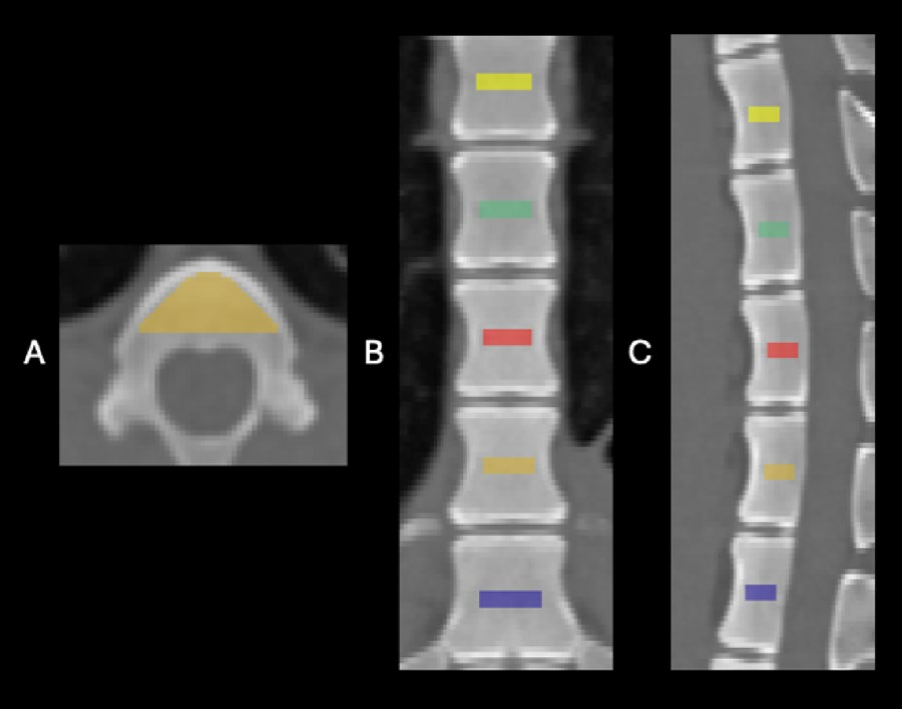
Placement of the regions of interest (ROI) in the vertebral bodies of a Göttingen minipig in a non-contrast CT examination with a radiation dose of 10.01 mGy in **A** axial, **B** coronal and **C** sagittal view. Th9 is annotated in brown. mGy = milligray.

In addition to fully automated segmentation of the complete vertebral body (trabecular and cortical bone), CT BMD measurement was performed using fully automated placed regions of interest (ROI). The ROI were placed in the center of each vertebral body in the axial, sagittal, and coronal planes. The cortical bone structure was explicitly avoided by cutting off three millimeters of the outer ROI boundary. The segmentation results were visually confirmed for each scan (Fig. 2).

### Control segmentation and validation of the automated BMD measurement

To assess the reliability of the nnU-network, manual segmentations were performed on three Göttingen minipigs under the supervision of a board-certified radiologist (ten years of experience), resulting in 450 manual annotated slices. Overlap of the fully automated segmentation of Th9 and the manual control was assessed by the Dice-Score, where a higher score reflects higher agreement^34^:

$$\:DICE\:\left(A,\:B\right)=\frac{2\times\:\:\mid\:A\cap\:B\mid\:}{\mid\:A\mid\:+\:\mid\:B\mid\:}$$

A and B represent the fully automatically generated (A) and manually segmented (B) set of images. $\:\mid\:A\mid\:$ & $\:\mid\:B\mid\:$ denote as the respective sample size, while $\:\mid\:A\cap\:B\mid\:$ stands for the size of the intersection between the two groups.

### Statistical analysis

The Shapiro-Wilk test was used to analyze the distribution of the data. Since the data in all groups were normally distributed (*p* > 0.05), Mauchly’s test for sphericity was applied. Since the assumption of sphericity was accepted (*p* > 0.05), a repeated measures analysis of variance (RM-ANOVA) was performed to determine statistical significance and test for a systematic dose effect across the repeated intraindividual measurements. The significance level was set at *p* = 0.05. Analyses were performed and displayed using IBM SPSS Statistics 31 (IBM, Armonk, New York, USA). Data are presented as mean and standard deviation in parentheses.

## Results

### Evaluation of the nnU-network

The Dice score for the overlap of fully automated segmentation of the complete ninth vertebra to manual controls was 0.961 (Dice_ovr_). The analysis of the subgroups yielded the following Dice Scores: Dice_5%_ = 0.942, Dice_10%_ = 0.960, Dice_20%_ = 0.969, Dice_40%_ = 0.976 and Dice_control_ = 0.959.

### BMD measurement of the complete vertebra

**Fig. 3:**
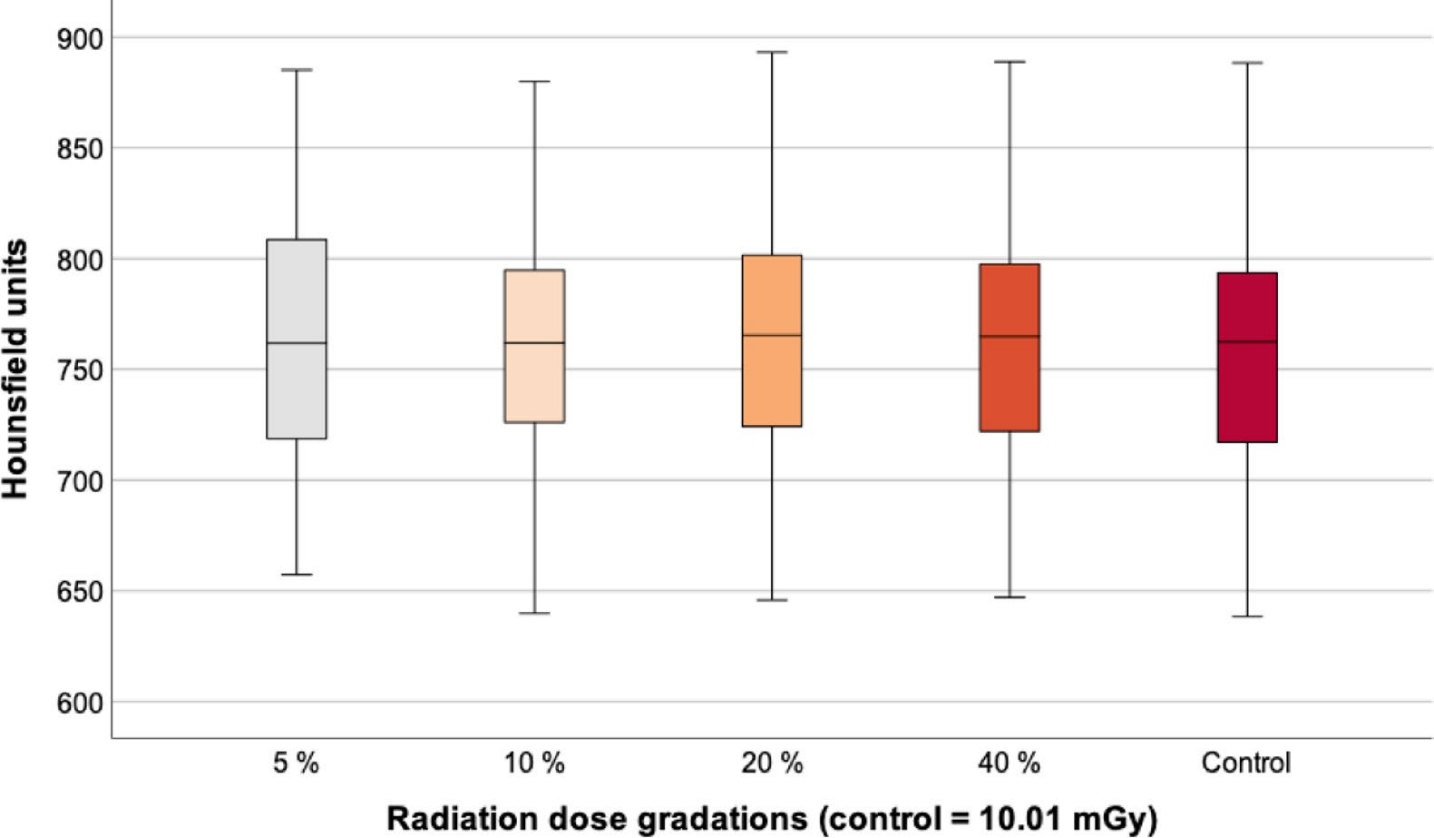
BMD measurement of the complete vertebra. There were no significant BMD differences between the control and the different radiation doses. BMD, bone mineral density; mGy, milligray.

BMD measurement did not differ significantly and therefore remained consistent under the different scan conditions. In fact, the BMD was almost the same in all dose gradations. Between the group with the lowest applied dose (5% = 761 [± 56] HU) and the control group (756 [± 55] HU) the RM-ANOVA did not show a significant difference (*p* = 0.956). There was also no significant difference between the control and the examinations with 10% (756 [± 55] HU, *p* = 1.0), with 20% (762 [± 55] HU, *p* = 0.915) and with 40% (759 [± 54] HU, *p* = 0.997). The differences in BMD measurements between the individual groups were minimal; for example, the mean value for the 5% group (761 [± 56] HU) was 0.7% higher than in the control group (756 [± 55] HU) (Table 2; Fig. 3).

**Table 2:** Results of the BMD measurement of the complete ninth vertebra.

Group	Mean (HU)	SD (HU)	Min. (HU)	Max. (HU)
5%	761	56	657	885
10%	756	55	640	880
20%	762	55	646	893
40%	759	54	647	889
Control	756	55	638	888

HU, Hounsfield unit; SD, standard deviation; Min., minimum; Max., maximum.

### BMD measurement by ROI placement

**Fig. 4:**
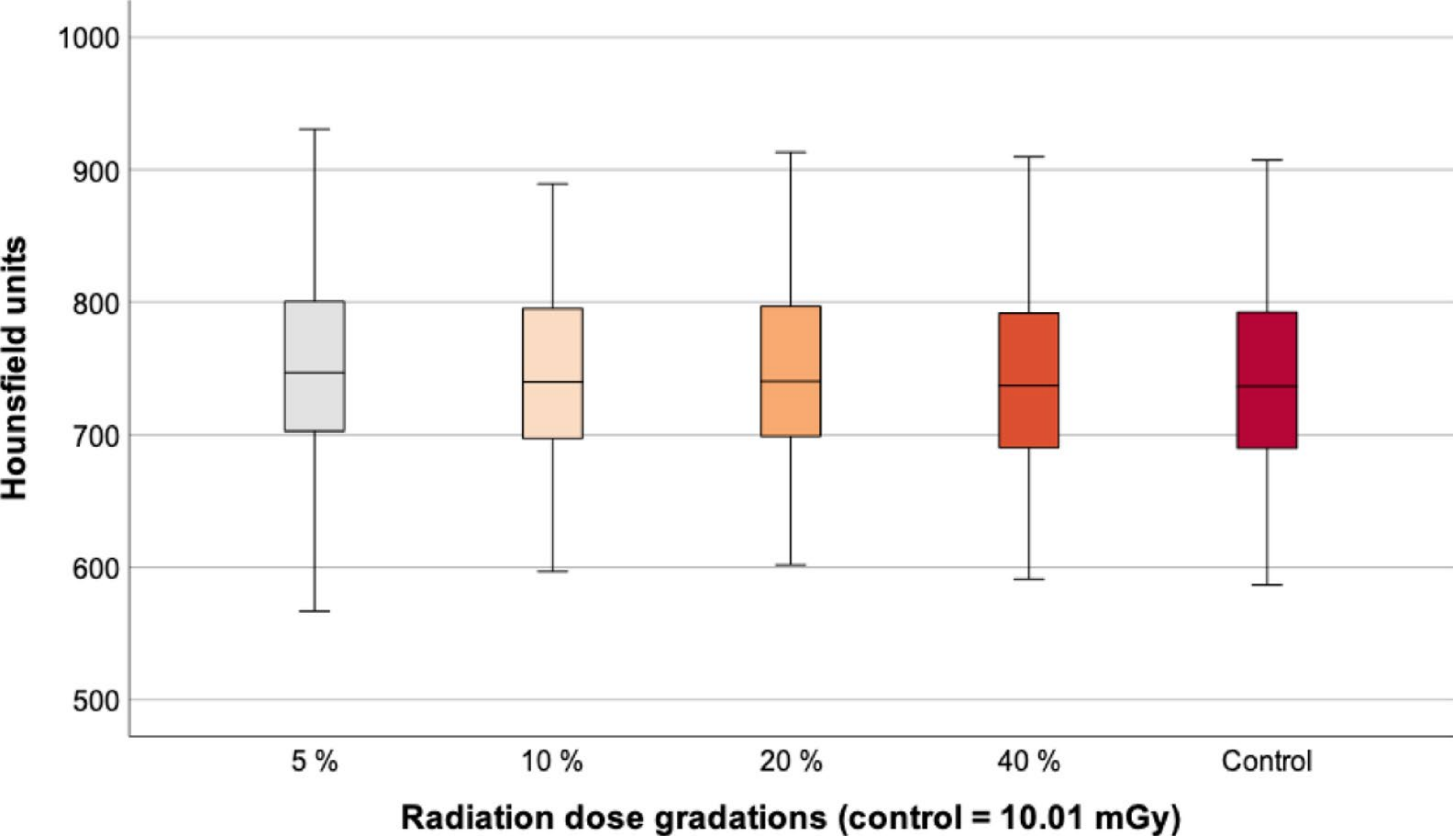
BMD measurement by placing ROIs. There were no significant BMD differences between the control and the different radiation doses. BMD, bone mineral density; mGy, milligray.

The CT based BMD measurement using ROI-placement also remained consistent between the control (738 [± 68] HU) and the examinations with 5% (749 [± 72] HU, *p* = 0.740), 10% (740 [± 68] HU, *p* = 0.999), 20% (745 [± 70] HU, *p* = 0.937) and 40% (739 [± 69] HU, *p* = 1.0). The relative deviation was minimal with a maximum difference of 1.5% between the control (738 [± 68] HU) and the 5% group (749 [± 72] HU) (Table 3; Fig. 4).

**Table 3:** Results of the BMD measurement by placing ROIs in the ninth thoracic vertebra.

Group	Mean (HU)	SD (HU)	Min. (HU)	Max. (HU)
5%	749	72	567	930
10%	740	68	597	889
20%	745	70	602	913
40%	739	69	591	910
Control	738	68	587	907

HU, Hounsfield unit; SD, standard deviation; Min., minimum; Max., maximum.

### Comparison of BMD measurement of the complete vertebra versus ROI placement

The BMD values were significantly lower using ROI placement compared to the segmentation of the complete ninth vertebral body (e.g., Control: 738 [± 68] HU versus 756 [± 55] HU, *p* = 0.047). For all values, refer to Tables 2 and 3.

## Discussion

The systematic investigation of the impact of radiation dose on the BMD measurement in an animal model reveals two major findings. First, the assessment of bone radiodensity as a surrogate for BMD remained consistent in different dose gradations. Second, radiodensity was significantly lower in the trabecular bone than in the entire vertebra. This underscores the potential applicability of CT-based BMD assessment in the trabecular bone within the clinical routine on non-contrast CT scans, not significantly affected by radiation dose.

To the best of our knowledge, no BCA network trained primarily for humans had yet been applied for CT BMD measurement in another species. In order to obtain valid results despite the anatomical differences between the vertebral bodies of Göttingen minipigs and humans, the BOA network was adjusted by manually training 65% of the animals examined^2^. Precise segmentation was ensured qualitatively by manual control performed by a board-certified radiologist (ten years of experience) and quantitatively by calculating the DICE score. A DICE score above 0.9 is considered to indicate excellent segmentation accuracy^35^. Since the DICE scores of all subgroups were above 0.94, this is the case in our study.

Previously, studies demonstrated consistent BMD measurements at significantly reduced radiation doses^22,30,36^. For instance, Zhang et al. utilized standard dose CT (120 kV, 250 mAs) and ULD-CT (100 kV, 30 mAs) which were conducted before and after lumbar spine surgery. Their phantomless CT-based BMD measurements via manual ROI placement yielded constant results in the standard and ULD scan. Still, the unclear influence of perioperative changes in the context of lumbar spine surgery and a mean time interval of 5.5 days are limitations to the study^30^.

In a phantom analysis, Li et al. showed consistent CT-based BMD measurement under the reduction of the radiation dose by 85% (normal: 200–370 mA, low-dose: 40 mA) using the European Spine Phantom^22^. The radiation dose was reduced by decreasing the tube current as we did in our study^22^. Although standardized conditions could be achieved using the phantom model, the restricted transferability of the phantom’s results to humans is a significant limitation.

To complement the latest research, this systematic large animal study compared the effect of reduced radiation dose in vivo with the elimination of disturbing elements in a controlled setting. We were able to underscore the consistency of CT-based BMD measurement under different dose conditions in repeated intraindividual scans. Both for the entire vertebral body and for a ROI in the trabecular bone, with only maximum mean deviations of 0.7% (complete) and 1.5% (ROI) in the 5% group compared to a control.

The comparison of CT-based BMD measurements between different dose gradations and the respective image quality is important because of the striking intra- and interinstitutional differences and the ongoing surge of low dose (LD) and ULD CT imaging^37,38^. Additionally, the comparison is relevant because of the steadily increasing number of countries employing lung cancer screening programs via LD-CT^39^.

Previous studies have already demonstrated, those scans offer a huge potential to gather information for opportunistic screening^24,40^. In a nationwide multicenter study in China involving 69.095 patients, Cheng et al. were able to identify a large number of patients with low BMD through the opportunistic use of low-dose chest CT scans obtained for lung cancer investigations^40^. Moreover, CT scans analyzed with advanced deep learning algorithms can detect subtle changes in bone density that DXA scans might overlook^24^.

Additionally, our study enables the longitudinal comparison of CT-based BMD measurements with different radiation doses to the extent that the controlled large animal model is transferable to humans. Even the BMD of the 5% and 10% group were comparable to the results of the control group and their CTDIvol was lower than the established reference values for cancer screening^41^. As the deviations of the remaining groups were not significant as well, this large animal study indicates first, that it is also possible to compare dedicated LD-CTs with each other, and second, that dose variations in the spectrum of regular full-dose examinations can be compared with each other. This might be relevant in clinical situations in which CT examinations with different protocols and correspondingly different radiation doses are to be used to assess the trend in BMD. Precise BMD monitoring during cortisone therapy in order to detect possible iatrogenic osteoporosis at an early stage is a potential use case.

BMD was previously measured in CT-examinations using a number of different methods. For example, Azekawa et al. manually placed ROIs in the spine^10^. Lou et al. manually examined the trabecular BMD at one level of the first lumbar vertebra^9^. A further development was the volumetric BMD measurement approach by Koch et al., in which the trabecular volume of interest (VOI) was determined manually for each vertebral body^42^. The advantage was the more accurate volumetric BMD measurement, avoiding the segmentation of the cortex. The main disadvantage was the high effort required for this method. In this study, BMD was extracted fully automatically using a machine learning algorithm. The primary benefit of this approach is that it enables opportunistic osteoporosis screening for a large number of patients while saving time and reducing costs.

In our study, BMD was determined using two methods. First, the complete vertebral body was segmented and second, only the trabecular bone of the vertebral body was segmented fully automatically using ROI placement. The reason for examining the complete vertebral body was based on the reference standard for BMD measurement to date, the DXA scan^18,43^.

To eliminate the influence of the cortical bone, as previously described in comparable studies, a ROI was automatically placed in the trabecular bone of the vertebra^36^. Compared to the segmentation of the entire vertebra, the CT-based BMD was significantly lower (e.g., Control: 738 [± 68] HU versus 756 [± 55] HU, *p* = 0.047). Therefore, measuring BMD with the same method is mandatory.

Some limitations need to be addressed. In our study, only non-contrast scans were assessed, and the influence of contrast medium application remains to be investigated. Another limitation was the reduced transferability because of anatomical differences in the vertebrae between Göttingen minipigs and humans. Moreover, the dose values from our large animal study are only partially transferable to humans due to differences in size and weight. The different object diameters between Göttingen minipigs and humans on image noise and CT-derived BMD measurements were not quantified in this study. Moreover, motion and positioning variability in the clinical routine may reduce the reproducibility of CT-derived BMD measurements compared with the standardized animal setup.

These limitations regarding direct transferability to humans were due to the fact that, it would not have been possible to conduct an experimental setup involving five intraindividual CT scans at different radiation doses, under otherwise identical scanning conditions and using the same scanner in a human cohort for ethical reasons. A further limitation is that all images were reconstructed using a single scanner-specific algorithm and kernel, which may limit generalizability to other reconstruction algorithms, scanner models, or vendors. Another limitation was that only the vertebral bodies of healthy Göttingen minipigs were examined. Furthermore, the assessment of BMD using CT-based radiodensity as a surrogate for absolute volumetric BMD is a limitation.

Finally, CT-based BMD assessment remained consistent across a wide range of radiation doses in this systematic large animal study. This indicates the technical feasibility of CT-based opportunistic BMD assessment and the possibility of a comparison between human CT scans with varying radiation doses.

## Data Availability

The datasets generated during and analyzed during the current study are available from the corresponding author on reasonable request.

## Abbreviations

%: Percent
AI: Artificial intelligence
BCA: Body composition analyses
BMD: Bone mineral density
BOA: Body and organ analyses
CT: Computed tomography
CTDIvol: Volumetric CT dose index
DXA: Dual energy X-ray absorptiometry
h: Hour
HU: Hounsfield unit
IQR: Interquartile range
kg: Kilogram
kV: Kilovolt
m^2^: Square meter
mAs: Milliampere seconds
mg: Milligram
mGy: Milligray
ML: Machine learning
ml: Milliliter
mm: Millimeter
NSCLC: Non-small cell lung cancer
qCT: Quantitative computed tomography
RM-ANOVA: Repeated measures analysis of variance
ROI: Region of interest
s: Second
ULD: Ultra-low dose
VOI: Volume of interest
